# Illustrated Lectures on Ambulance Work

**Published:** 1889-03

**Authors:** 


*Illustrated Lectures on Ambulance Work.* By R. LAWTON ROBERTS, M.D. Pp. 206. London: H. K. LEWIS. 1888.

We have nothing that is not favourable to say respecting this little work. It seems very satisfactorily to fulfil the commendable object with which it was written; namely, to instruct the laity how best to deal with cases of emergency occurring in their midst. As a text-book to be used in the classes formed by the St. John's or other Ambulance Associations, it is sure to be popular and useful.

